# Urine S100 proteins as potential biomarkers of lupus nephritis activity

**DOI:** 10.1186/s13075-017-1444-4

**Published:** 2017-10-24

**Authors:** Jessica L. Turnier, Ndate Fall, Sherry Thornton, David Witte, Michael R. Bennett, Simone Appenzeller, Marisa S. Klein-Gitelman, Alexei A. Grom, Hermine I. Brunner

**Affiliations:** 1 Department of Rheumatology, Cincinnati Children’s Hospital Medical Center, MLC 4010, 3333 Burnet Avenue, Cincinnati, OH 45229 USA; 2 Department of Pathology and Laboratory Medicine, Cincinnati Children’s Hospital Medical Center, 3333 Burnet Ave, Cincinnati, OH 45229 USA; 3 Department of Nephrology, Cincinnati Children’s Hospital Medical Center, 3333 Burnet Ave, Cincinnati, OH 45229 USA; 4 State University of Campinas, Barão Geraldo, Campinas, SP 13083-970 Brazil; 5 Ann & Robert H. Lurie Children’s Hospital of Chicago, 225 East Chicago Avenue, Chicago, IL 60611 USA

**Keywords:** Systemic lupus erythematosus, Lupus nephritis, Biomarker, S100 protein, S100A4

## Abstract

**Background:**

Improved, noninvasive biomarkers are needed to accurately detect lupus nephritis (LN) activity. The purpose of this study was to evaluate five S100 proteins (S100A4, S100A6, S100A8/9, and S100A12) in both serum and urine as potential biomarkers of global and renal system-specific disease activity in childhood-onset systemic lupus erythematosus (cSLE).

**Methods:**

In this multicenter study, S100 proteins were measured in the serum and urine of four cSLE cohorts and healthy control subjects using commercial enzyme-linked immunosorbent assays. Patients were divided into cohorts on the basis of biospecimen availability: (1) longitudinal serum, (2) longitudinal urine, (3) cross-sectional serum, and (4) cross-sectional urine. Global and renal disease activity were defined using the Systemic Lupus Erythematosus Disease Activity Index 2000 (SLEDAI-2K) and the SLEDAI-2K renal domain score. Nonparametric testing was used for statistical analysis, including the Wilcoxon signed-rank test, Kruskal-Wallis test, Mann-Whitney *U* test, and Spearman’s rank correlation coefficient.

**Results:**

All urine S100 proteins were elevated in patients with active LN compared with patients with active extrarenal disease and healthy control subjects. All urine S100 protein levels decreased with LN improvement, with S100A4 demonstrating the most significant decrease. Urine S100A4 levels were also higher with proliferative LN than with membranous LN. S100A4 staining in the kidney localized to mononuclear cells, podocytes, and distal tubular epithelial cells. Regardless of the S100 protein tested, serum levels did not change with cSLE improvement.

**Conclusions:**

Higher urine S100 levels are associated with increased LN activity in cSLE, whereas serum S100 levels do not correlate with disease activity. Urine S100A4 shows the most promise as an LN activity biomarker, given its pronounced decrease with LN improvement, isolated elevation in urine, and positive staining in resident renal cells.

**Electronic supplementary material:**

The online version of this article (doi:10.1186/s13075-017-1444-4) contains supplementary material, which is available to authorized users.

## Background

Childhood-onset systemic lupus erythematosus (cSLE) is a complex inflammatory autoimmune disease of unknown etiology that carries a risk of irreversible damage in multiple organ systems. Up to 80% of children with cSLE will develop lupus nephritis (LN), and LN persists as a leading cause of morbidity and mortality in patients with cSLE [1–4]. Current noninvasive laboratory and clinical measures of LN activity are not sensitive or specific enough to reliably measure the course of LN [5, 6], supporting the need for novel noninvasive biomarkers. S100 proteins have the potential to fill this unmet need.

S100 proteins are emerging as reliable biomarkers of disease activity in a multitude of pediatric inflammatory diseases, including systemic juvenile idiopathic arthritis and inflammatory bowel disease [7, 8]. These proteins represent a diverse group of calcium-binding proteins, and mainly the heterodimer S100A8/9 (also known as *MRP8/14* or *calprotectin*) and S100A12 have been studied to date in inflammatory diseases. S100A8/9 and S100A12 are expressed predominantly by phagocytes and serve as danger or damage-associated molecular patterns upon extracellular release, triggering continued inflammation [7].

Elevated serum levels of S100A8/A9 have been described in several adult SLE populations and correlate with disease activity [9, 10]. In particular, S100A8/9 expression is increased on interferon-producing plasmacytoid dendritic cells of patients with SLE with active disease [11]. Intrarenal S100A8/9 messenger RNA (mRNA) levels were also recently shown to predict progression to chronic kidney disease in an adult LN cohort [12].

Whereas S100A8/9 and S100A12 are predominantly phagocyte-specific proteins, S100A4 (also known as *fibroblast-specific protein 1* or *metastasin*) is more widely expressed and has demonstrated correlation with disease activity in various forms of glomerulonephritis [13]. S100A4 also has proposed roles in enhancement of cell motility, dysregulation of cell death, and progression of fibrosis that may be important for pathogenesis of LN and SLE in general [14, 15]. Similarly to S100A4, S100A6 functions to influence cell motility through cytoskeletal changes and can cause dysregulated apoptosis, which is known to be a contributor to development of autoimmunity in SLE.

Combined assessment of select S100 proteins in both serum and urine in relation to the status and course of cSLE has not been reported and was the focus of this study. The purpose of this study was to evaluate five S100 proteins (S100A4, S100A6, S100A8/9, and S100A12) for their relationship to renal and extrarenal cSLE activity. We hypothesized that serum S100 proteins would correlate with cSLE disease activity in general and that urine S100 levels would reflect LN activity.

## Methods

### Study population

For this study, we used banked serum and urine samples. For the purpose of analysis, four groups were defined:

1. *Longitudinal serum (Cohort L*_*s*_ *) (n = 47)*: Patients had serum samples collected at two time points, once during an active disease visit and once at an improved visit.
2. *Longitudinal urine (Cohort L*_*u*_ *) (n = 39)*: Patients had urine samples collected at the time of active LN and also at the time of improved LN.
3. *Cross-sectional serum (Cohort X*_*s*_ *) (n = 100)*: Patients had a single serum sample collected from visits with active extrarenal disease activity only (*n* = 34), active LN (*n* = 41), or low global disease activity (*n* = 25).
4. *Cross-sectional urine (Cohort X*_*u*_ *) (n = 96)*: Patients had a single urine sample collected from visits with active extrarenal disease activity only (*n* = 18), active LN (*n* = 52), or low global disease activity (*n* = 26).

The samples from active disease visits of patients in Cohorts L_s_ and L_u_ were also included in the respective cross-sectional cohorts. Patients could also be included in both the serum and urine longitudinal cohorts if both serum and urine from an active and improved disease visit were present.

This study was approved by the Cincinnati Children’s Institutional Review Board with a waiver of consent and included patients from nine pediatric rheumatology centers. All patients met American College of Rheumatology classification criteria for SLE [16, 17] and had disease onset prior to 18 years of age. Patients were excluded if an active infection was present at the time of the visit, because infection may elevate S100 levels and become a potential confounder. Control serum and urine samples were obtained from healthy children enrolled in the Cincinnati Genomic Control Cohort [18, 19].

### Assessment of disease activity

For patients with cSLE, both extrarenal and renal disease activity were assessed using the Systemic Lupus Erythematosus Disease Activity Index 2000 (SLEDAI-2K) [20]. The SLEDAI-2K is a validated disease activity measure for cSLE with a total score of 0–105, consisting of 24 weighted items grouped into organ-specific domains. The SLEDAI-2K renal domain (SLEDAI-R) score incorporates four items: urinary casts (heme granular or red blood cell), hematuria (>5 red blood cells/high-power field), proteinuria (>0.5 g/24 h), and pyuria (>5 white blood cells/high-power field, excluding infection). Each item is scored a 4 if present, leading to a range of 0–16 with 0 indicating no LN activity [20]. The gold standard for diagnosis and monitoring of LN activity is a renal biopsy, with findings interpreted as suggested by the 2003 International Society of Nephrology/Renal Pathology Society (ISN/RPS) classification [21]. Active features of inflammation on biopsy are classified by the National Institutes of Health activity index (NIH-AI), with a range of 0–24 and 0 signifying no active LN features [22].

In this study, an active disease visit was defined by a SLEDAI-2K score ≥ 8, and an improvement of cSLE was defined by a decrease in SLEDAI-2K score by ≥ 6. A low global disease activity visit was defined by an extrarenal SLEDAI-2K score ≤ 4. Those patients with active disease were classified as having active LN if they had a SLEDAI-R score ≥ 4, and an improvement in LN was defined by a decrease in SLEDAI-R score by ≥ 4.

### Clinical data collection

Demographic and clinical information was collected on all patients at the time of visits, including age at time of diagnosis, disease duration, global SLEDAI-2K score and individual organ-specific disease scores, laboratory data, current medications, blood pressure, and estimated glomerular filtration rate (eGFR; calculated using the original Schwartz method) [23, 24]. Anti-double-stranded (ds)DNA status was categorized by positivity or negativity. More detailed information was additionally collected regarding LN history in Cohorts L_u_ and X_u_, including ISN/RPS class of LN [21], date of renal biopsy, and NIH-AI on biopsy.

### S100 proteins in serum and urine

Serum was obtained from whole blood collected in serum separator tubes and stored frozen at − 80 °C. Urine was also collected and stored frozen at − 80 °C.

S100 protein levels were measured in duplicate in the serum and urine with commercially available enzyme-linked immunosorbent assays (BÜHLMANN MRP8/14; BÜHLMANN Laboratories, Schönenbuch, Switzerland; CircuLex S100A12/EN-RAGE, CircuLex S100A4, and CircuLex S100A6; MBL International, Nagano, Japan) per the manufacturers’ instructions.

### S100A4 immunohistochemistry

Immunohistochemistry (IHC) was performed using 4-μm sections from four paraffin-embedded LN biopsy specimens and two control specimens from 21 and 28 week gestation kidneys. A polyclonal rabbit antihuman S100A4 antibody was used at a 1:75 dilution as per the manufacturer’s instructions (Dako, Carpinteria, CA, USA). The stains were run on the Roche BenchMark ULTRA instrument (Ventana Medical Systems, Tucson, AZ, USA) prior to the antibody (incubated for 32 minutes) and pretreated with Roche Mild Cell Conditioning 2 (CC2, citrate; Ventana Medical Systems). The slides were then detected with the Roche ultraView detection kit and counterstained with Roche Hematoxylin and Bluing Reagent (Ventana Medical Systems).

### Statistical analysis

Statistical analysis was performed using Prism version 7.01 (GraphPad Software, La Jolla, CA, USA) and Excel 2016 (Microsoft, Redmond, WA, USA) software. Median values and IQRs of continuous variables and number/percent of categorical values were calculated as descriptive statistics. Patient samples with a nondetectable S100 value were assigned a value of 0 for the purpose of statistical analysis. Serum and urine S100 levels were analyzed using nonparametric testing given a nonnormal distribution of S100 values. Both nonnormalized urine S100 values and urine S100 values normalized to the visit urine creatinine were evaluated. Raw urine S100 values are reported in the Results section below because analyses were unchanged with normalized S100 values.

For analysis of the change in S100 and laboratory values between paired samples in Cohorts L_s_ and L_u_, the Wilcoxon signed-rank test was applied. The Kruskal-Wallis test and Mann-Whitney *U* test were used to evaluate for differences between S100 levels among patient groups in Cohorts X_s_ and X_u_ and also between patients with different clinical categorizations in all active samples. Extrarenal disease activity was not corrected for in patients with active LN. Spearman’s rank correlation coefficients (*r*_s_) were calculated to assess the relationships between different S100 protein levels, SLEDAI-2K scores, SLEDAI-R scores, and standard laboratory tests.

## Results

### Clinical and demographic data for all cohorts

Additional file 1: Table S1 summarizes patient characteristics from Cohorts X_s_ and X_u_. Patients in both cohorts had a similar age at diagnosis, with a median of 13–15 years. Overall, patients in Cohort X_s_ had longer disease duration at the time of sample collection than patients in Cohort X_u_ (5–8 years compared with 2–2.5 years). In both cohorts, the total SLEDAI-2K score was 12–13 for active visits and 2 for low disease activity visits. No patients had active renal involvement in the low disease activity groups of either cohort, as per the given disease activity definition.

Table 1 provides a summary of the patient characteristics from active visits in Cohorts L_s_ and L_u_. Both cohorts were diverse and had serologically active disease. Similar to Cohorts X_s_ and X_u_, the median disease duration was 3 years at the time of urine sample collection and 5 years at serum sample collection. The median time between active and improved visits in Cohort L_u_ was 16 months (IQR 7–28), and that for Cohort L_s_ was 11 months (IQR 6–21.5). The majority of patients in both cohorts had both extrarenal and renal involvement. Nearly all patients had biopsy-proven LN in Cohort L_u_, with the exception of two patients. The median SLEDAI-R score for an active and improved LN visit in Cohort L_u_ was 12 (IQR 8–12) and 0, respectively. Only 2 of the 39 patients in Cohort L_u_ had an eGFR < 75 ml/minute/1.73 m^2^.

**Table 1:** Demographic and clinical data on patients with childhood-onset systemic lupus erythematosus from Cohorts L_s_ and L_u_

	Cohort L_s_ (*n* = 47)	Cohort L_u_ (*n* = 39)
Characteristic	Active SLE serum	Active LN urine
Age at diagnosis, years	15 (10.5–17.5)	14 (12–16)
Disease duration, years	5 (3–9)	3 (1–4.5)
Female sex, *n* (%)	41 (87.2)	31 (79.5)
White race, *n* (%)	27 (57.4)	18 (46.2)
Hispanic ethnicity, *n* (%)	18 (38.3)	6 (15.4)
Total SLEDAI-2K score	13 (10–17)	17 (12–20)
Active renal involvement, *n* (%)	24 (51.1)	39 (100)
Biopsy-proven, *n* (%)	14^a^ (93.3)	37 (94.9)
SLEDAI-R score	8 (4–12)	12 (8–12)
ISN/RPS class of LN, III/IV/V, *n* (%)	3/9/6^a^ (21.4/64.3/42.9)	5/16/12 (13.5/43.2/32.4)
eGFR < 75 ml/minute/1.73 m^2^, *n* (%)	1^a^ (3.3)	2 (5.1)
Active extrarenal involvement, *n* (%)	46 (97.9)	38 (97.4)
Medications, *n* (%)		
Oral or intravenous steroids	40 (85.1)	35 (89.7)
Hydroxychloroquine	41 (87.2)	32 (82)
Other immunosuppressant	26 (55.3)	27 (69.2)
Laboratory tests		
Positive anti-dsDNA, *n* (%)	18^a^ (60)	31 (79.5)
C3, mg/dl	83.6^a^ (59.5–114.8)	65 (45.4–93.3)
C4, mg/dl	10.6^a^ (7–16.2)	8.6 (4.5–11.1)
Random urine protein/creatinine	0.26^a^ (0.12–1.8)	2.1 (1.1–5.3)
Active urinary sediment, *n* (%)	14^a^ (46.7)	32 (82.1)

*Abbreviations: Cohort L*_*s*_ Longitudinal serum cohort, *Cohort L*_*u*_ Longitudinal urine cohort, *eGFR* Estimated glomerular filtration rate, *ISN/RPS* International Society of Nephrology/Renal Pathology Society, *LN* Lupus nephritis, *SLEDAI-2K* Systemic Lupus Erythematosus Disease Activity Index 2000, *SLEDAI-R* Systemic Lupus Erythematosus Disease Activity Index 2000 renal domain score, *SLE* Systemic lupus erythematosus

All table values are expressed as median (IQR) for continuous variables and number (percent) for categorical variables

^a^Note that certain data only available for SLE patients with serum from Cincinnati Children’s Hospital Medical Center (*n* = 30), not Brazilian patients (*n* = 17). Percentages in table were calculated from patients with available values for these characteristics

### Serum S100 levels in patients with cSLE

**Fig. 1:**
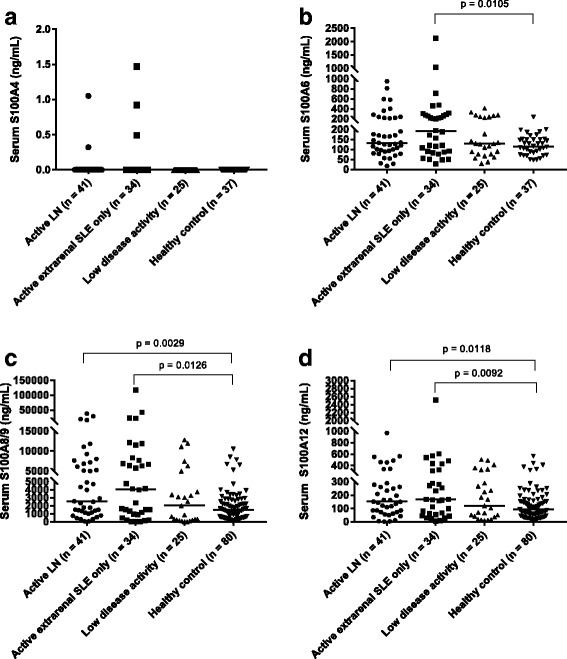
Comparison of serum S100 levels in patients with childhood-onset systemic lupus erythematosus (cSLE) from the cross-sectional serum cohort, including those with active lupus nephritis (LN), active extrarenal SLE only, and low disease activity, as well as healthy control subjects. **a** Serum S100A4. **b** Serum S100A6. **c** Serum S100A8/9. **d** Serum S100A12. The *horizontal lines* on each plot represent median S100 values. Differences in serum S100 levels between patient groups were assessed using the Kruskal-Wallis and Mann-Whitney *U* tests

**Fig. 2:**
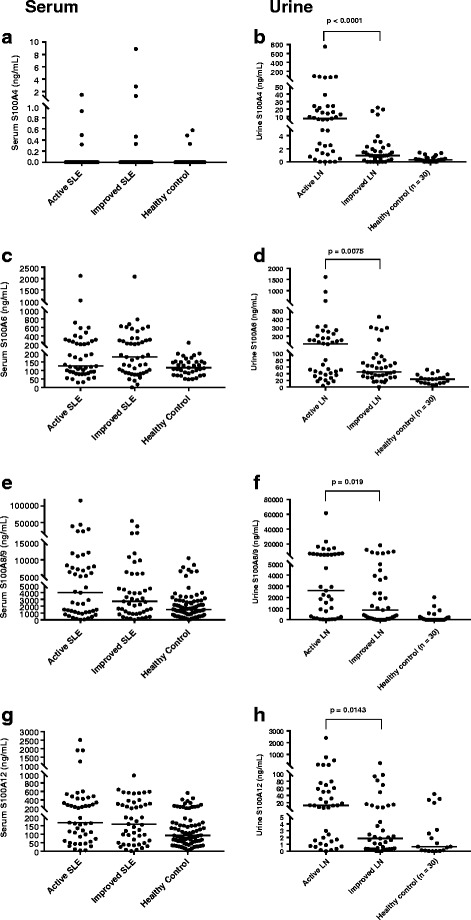
Serum and urine S100 levels in patients with childhood-onset systemic lupus erythematosus (cSLE) from longitudinal serum and urine cohorts. **a** Serum S100A4. **b** Urine S100A4. **c** Serum S100A6. **d** Urine S100A6. **e** Serum S100A8/9. **f** Urine S100A8/9. **g** Serum S100A12. **h** Urine S100A12. The *horizontal line* represents the median S100 value on each plot. The Wilcoxon signed-rank test was used to assess for a significant change between active and improved LN visits

We observed no differences in any of the serum S100 levels when comparing cSLE patients with active LN, active extrarenal SLE only, or low disease activity (Fig. 1). Serum S100A6, S100A8/9, and S100A12 levels were all higher in patients with active extrarenal SLE than in healthy control subjects (Fig. 1b–d). Serum S100A8/9 and S100A12 levels were also higher in patients with active LN than in healthy control subjects (Fig. 1c, d). Patients with cSLE with low disease activity had serum S100A6, S100A8/9, and S100A12 levels comparable to those of healthy control subjects. Serum S100A4 levels were detectable in only 5 of the 100 cSLE patient samples tested and were undetectable in healthy control subjects. Serum S100 levels were similarly not found to differ between active and improved visits of patients with cSLE, regardless of SLE (extrarenal, renal) disease activity (Fig. 2a, c, e, g).

### Urine S100 levels in patients with cSLE with and without LN from cross-sectional cohort

**Fig. 3:**
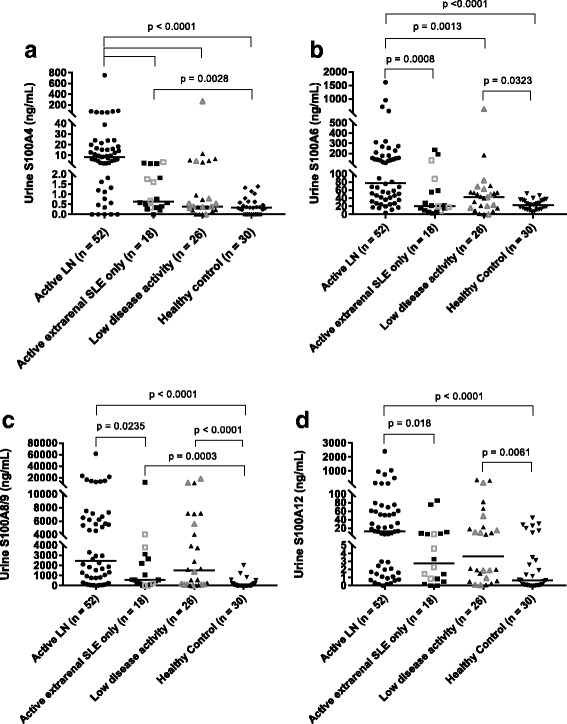
Comparison of urine S100 levels in patients with childhood-onset systemic lupus erythematosus (cSLE) from cross-sectional urine cohort, including those with active lupus nephritis (LN), active extrarenal SLE only, and low disease activity, as well as healthy control subjects. (**a** Urine S100A4. **b** Urine S100A6. **c** Urine S100A8/9. **d** Urine S100A12. Patients in the active extrarenal SLE only and low disease activity categorizations at the time of sample collection with a prior history of LN are signified by *open symbols*. The *horizontal line* on each plot represents the median S100 value. Note that the scale of the *y*-axis is broken to allow visualization of all patient values. Differences in urine S100 levels between patient groups were assessed using the Kruskal-Wallis and Mann-Whitney *U* tests

Urine levels of all tested S100 proteins were higher with active LN than with active extrarenal SLE only (Fig. 3). Differences in urine levels between active renal versus active extrarenal SLE only were most pronounced for S100A4 level (median [IQR] 8.35 ng/ml [1.7–19.4] in active LN versus 0.63 ng/ml [0.3–1.9] in active extrarenal SLE only; *p* < 0.0001) (Fig. 3a). In urine samples, only S100A4 and S100A6 levels differed between patients with active LN and patients with cSLE with low disease activity, whereas urine S100A8/9 and S100A12 levels trended toward significance. All urine S100 levels in Cohort X_u_ were higher in patients with active LN than in healthy control subjects. Urine S100A6, S100A8/9, and S100A12 levels were all also higher in patients with cSLE with low disease activity than in healthy control subjects. Urine S100A4 levels were comparable between patients with cSLE with low disease activity and healthy control subjects.

### Urine S100 levels in patients with cSLE with LN from longitudinal cohort

All urine S100 levels differed significantly between active and improved visits in patients with cSLE with LN. Figure 2 demonstrates urine S100 values in the paired samples from Cohort L_u_, with healthy control subjects also depicted for comparison. Refer to Additional file 2: Table S2 for urine S100 values in patients with cSLE from active and improved visits.

With improvement of LN (active versus improved visit), urine S100 levels decreased (Fig. 2 b, d, f, h). The most pronounced difference with changing LN activity was observed for urine S100A4 levels (median change 6.38 ng/ml; *p* < 0.0001) (Fig. 2b). The median urine S100A4 levels were 7.35 ng/ml (IQR 1.6–23.9) for active LN visits, 0.97 ng/ml (IQR 0.4–2.2) for improved LN visits, and 0.32 ng/ml (IQR 0–0.6) for healthy control subjects.

### Urine S100A4 levels in all patients with active LN by LN class and degree of LN activity

**Fig. 4:**
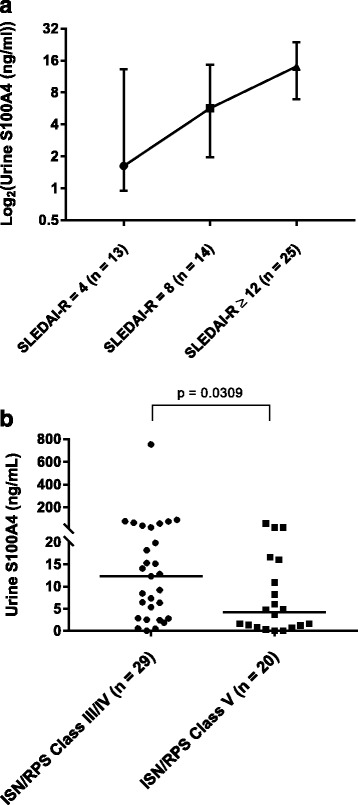
Urine S100A4 levels from all patients with active lupus nephritis (LN) in longitudinal urine and cross-sectional urine cohorts compared according to (**a**) LN activity as defined by Systemic Lupus Erythematosus Disease Activity Index 2000 renal domain (SLEDAI-R) score and (**b**) International Society of Nephrology/Renal Pathology Society (ISN/RPS) class of LN. **a** The median and IQR S100A4 levels are depicted. The median urine S100A4 level for a SLEDAI-R score of 4 was 1.62 ng/ml (IQR 0.95–13.2), increasing to 5.68 ng/ml (IQR 1.97–14.6) for a SLEDAI-R score of 8 and 14.08 ng/ml (IQR 6.9–23.97) for a SLEDAI-R score ≥ 12. A statistically significant difference was present between patients with LN with a SLEDAI-R score of 4 and those with a SLEDAI-R score ≥ 12 (*p* = 0.032). **b** The median is represented by the *horizontal line* on each plot. Differences between urine S1004 values in class III/IV versus class V LN were assessed using the Mann-Whitney *U* test

Urine S100A4 levels were higher with increased levels of LN activity (SLEDAI-R) (Fig. 4a). The median urine S100A4 level for a SLEDAI-R score of 4 was 1.62 ng/ml (IQR 0.95–13.2), for a SLEDAI-R score of 8 it was 5.68 ng/ml (IQR 1.97–14.6), and for a SLEDAI-R score of ≥ 12 it was 14.08 ng/ml (IQR 6.9–23.97). A statistically significant difference was present between patients with LN with a SLEDAI-R score of 4 versus those with a SLEDAI-R score ≥ 12 (*p* = 0.032 by Mann-Whitney *U* test). Urine S100A4 levels also differed among ISN/RPS classes of LN (Fig. 4b). Median S100A4 urine levels were significantly higher among those with class III/IV LN than among those with class V LN (median [IQR] 12.3 ng/ml [2.8–31.5] versus 4.2 ng/ml [0.89–58.4]; *p* = 0.03).

For those patients with LN with renal biopsies done within 2 months of urine sample collection (*n* = 25), there was no statistically significant association of urine S100A4 levels with NIH-AI (*r*_s_ = 0.18, *p* = 0.4). Likewise, no association was observed between urine S100A6, S100A8/9, or S100A12 level and NIH-AI.

### Association of urine S100 levels with clinical disease activity measures and other S100 protein levels in all patients with active LN

All urine S100 protein levels had a weak positive association with SLEDAI-R score in patients with active LN (for S100A4, *r*_s_ = 0.34, *p* = 0.014; for S100A6, *r*_s_ = 0.22, *p* = 0.12; for S100A8/9, *r*_s_ = 0.31, *p* = 0.03; for S100A12, *r*_s_ = 0.4, *p* = 0.003). Urine S100A4 levels showed a moderate correlation with urine S100A6 (*r*_s_ = 0.46, *p* = 0.001), urine S100A8/9 (*r*_s_ = 0.47, *p* = 4.3 × 10^−4^) and urine S100A12 levels (*r*_s_ = 0.55, *p* = 2.2 × 10^−5^).

### Association of urine S100A4 levels with standard laboratory measures of disease activity in all patients with active LN

Urine S100A4 levels showed a weak negative correlation with both C3 and C4 levels (C3, *r*_s_ = −0.28, *p* = 0.05; C4, *r*_s_ = −0.32, *p* = 0.03) but lacked association with proteinuria (*r*_s_ = 0.08, *p* = 0.57). There was no difference in urine S100A4 levels based on anti-dsDNA positivity or negativity.

### S100A4 immunohistochemistry in renal biopsies of patients with LN versus control subjects

**Fig. 5:**
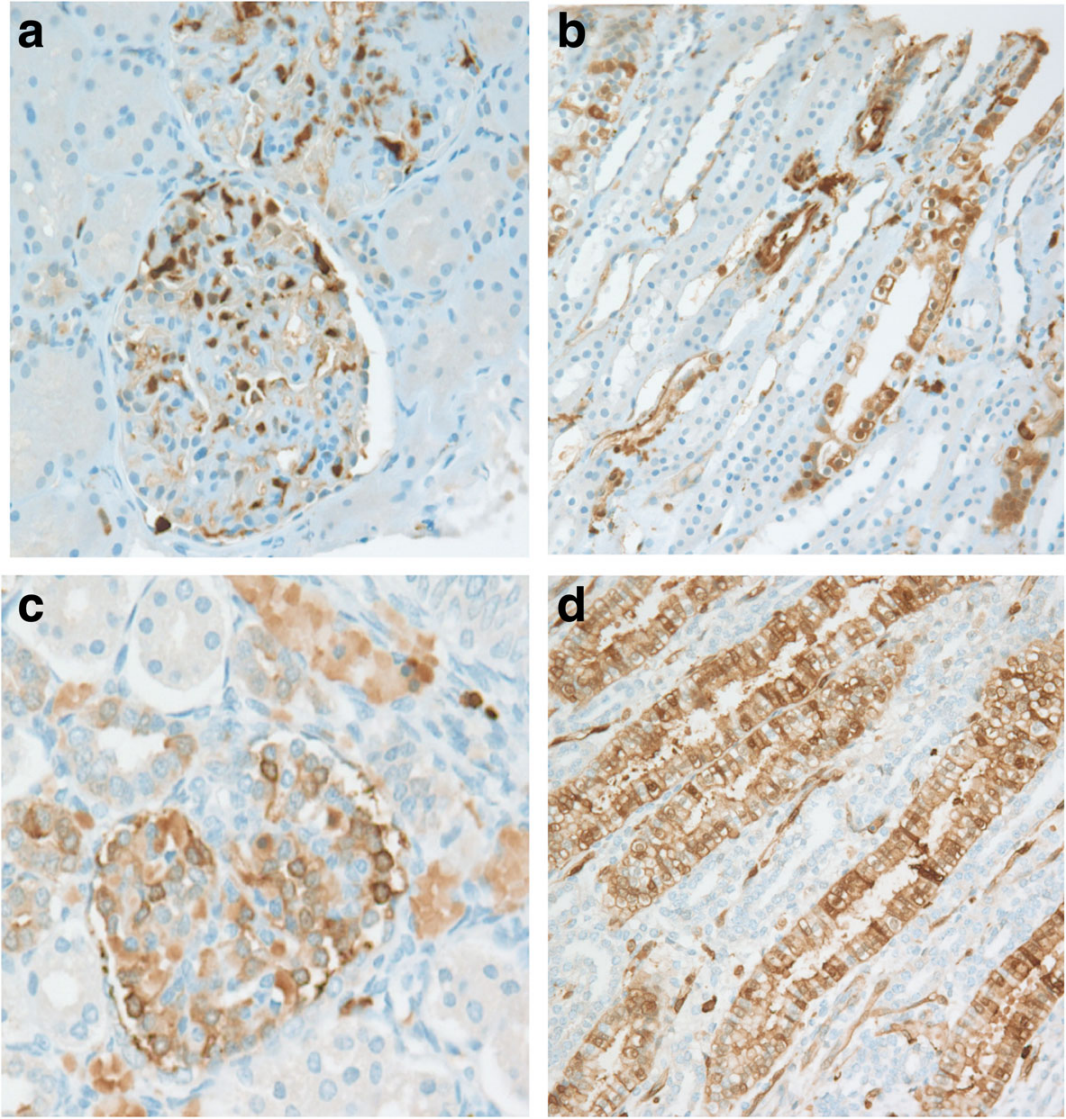
S100A4 immunohistochemistry in lupus nephritis (LN) and control biopsy specimens. LN kidney with nuclear and cytoplasmic staining in (**a**) podocytes and mononuclear cells, (**b**) distal tubular epithelial cells and control kidney with nuclear and cytoplasmic staining in (**c**) podocytes and mononuclear cells, and (**d**) distal tubular epithelial cells. Original magnification × 20

Both patients with LN and control subjects demonstrated positive nuclear and cytoplasmic S100A4 staining in renal biopsy specimens (Fig. 5). Staining was present in podocytes, mononuclear cells, and distal tubular epithelial cells of both LN and control biopsies. Whereas LN biopsy specimens had a moderate S100A4-positive mononuclear cell infiltrate, control specimens had only rare S100A4-positive mononuclear cells. The mononuclear cell staining pattern was primarily intranuclear. Proximal tubular epithelial cells and mesangial cells were predominantly negative.

## Discussion

We found elevated levels of urine S100 proteins with active LN, and urine S100 levels also demonstrated responsiveness to change in LN activity, with S100A4 showing the most pronounced differences. Conversely, S100 levels in the serum did not correlate with extrarenal or renal disease activity in cSLE, although serum S100A8/9 and S100A12 levels were higher in patients with active SLE than in healthy control subjects. To determine expression of S100A4 locally in the kidney, IHC was performed, which demonstrated positive staining for S100A4 in inflamed LN kidneys with expression in podocytes, distal tubular epithelial cells, and mononuclear cells.

S100 proteins represent a heterogeneous group of calcium-binding proteins that exhibit diverse functions and cell-specific expression patterns. In particular, S100A4 has been proposed to have roles in enhancing cell motility, promoting cell growth, mediating epithelial-mesenchymal transition (EMT), inhibiting cell death, and influencing the progression of fibrosis [14, 15]. Although S100A4 is well known in the oncology literature as a marker of metastatic tumor progression, it has not been studied as extensively in autoimmune diseases. Research to date, however, does support that S100A4 has both biologic plausibility as a biomarker of LN and a potential role in disease pathogenesis.

Urine S100A4 levels have previously been described to correlate with disease activity in various forms of crescentic glomerulonephritis [13]. S100A4 has also shown prognostic value as a biomarker in immunoglobulin A nephropathy, with the number of S100A4-positive cells on biopsy found to predict renal failure and corticosteroid responsiveness [25, 26]. Although prior data on S100A4 in patients with SLE are lacking, S100A4 mRNA was found to be upregulated in microdissected glomeruli of an MRL/lpr mouse model of LN [27].

The presence of positive S100A4 protein staining in resident renal cells further establishes biologic relevance for this novel biomarker of LN activity. IHC results from this study are in line with those of prior studies localizing S100A4 staining to podocytes and cellular crescents in antineutrophil cytoplasmic antibody-associated glomerulonephritis [13]. Given that IHC did not demonstrate a distinct differential S100A4 expression pattern between LN kidneys and those of control subjects, it remains undefined which cell type upregulates S100A4 expression in LN. One possibility is that resident renal cells increase production of S100A4 during a diseased state such as LN, with S100A4 levels increasing proportionally to LN activity. The isolated elevation of S100A4 in LN urine and lack of correlation with extrarenal disease activity or proteinuria lend support to this theory. In fact, a prior study demonstrated S100A4 expression in 86% of detached urine podocytes detected in patients with diabetic nephropathy [28]. Given the proposed role of S100A4 in EMT, S100A4 may play a role in dedifferentiation of podocytes prior to their detachment and loss in the urine.

Alternatively, S100A4 may be upregulated in inflammatory cells in LN, more in line with the known expression patterns of S100A8/9 and S100A12. In fact, biopsy specimens of patients with LN were found to have a higher number of S100A4-positive mononuclear cells than those of control subjects. S100A8/9 protein expression has been shown to correlate with inflammatory activity on renal biopsy in patients with varied etiologies of nephritis [29]. This does not necessarily equate to increased inflammatory cell infiltration. In fact, studies on S100A8/9 in glomerulonephritis suggest that increased S100A8/9 expression actually reflects a change in the immune cell phenotype versus a simple increase in the number of infiltrating immune cells within the kidney [29]. S100A4 may also fundamentally alter the behavior of immune cells central to the pathogenesis of LN.

Although S100 proteins have been studied predominantly in the serum, urine S100A8/9 has been shown to distinguish between intrinsic and prerenal acute kidney injury and even to be superior to neutrophil gelatinase-associated lipocalin (NGAL), the principal biomarker currently used for this assessment [30, 31]. We have shown that NGAL is a predictive biomarker of LN flares [32], and additional studies are needed to assess whether S100A8/9 or other S100 proteins are also predictive of LN flares.

Given the close relationship between the urine levels of the S100 proteins studied, we hypothesize that there is some redundancy between S100 protein functions and that most inflammatory cells, including neutrophils, express various S100 proteins. S100 proteins are components of neutrophil extracellular traps (NETs) [33], and NETosis contributes to type I interferon production in SLE and is associated with the presence of LN [34]. Moreover, gene expression signatures from peripheral blood leukocytes of patients with SLE include granulocyte-specific genes in addition to interferon-associated genes [35], lending further support to the theory that a neutrophil-specific protein such as an S100 protein family member could serve as an LN biomarker.

S100 proteins also possess some practical properties of an ideal biomarker. S100 proteins seem to be quite stable for measurement, both in serum and in urine, even after prolonged storage, in line with prior findings for NGAL [36]. Several studies have also documented that immunosuppressive drugs do not affect S100A8/9 expression [11, 29].

One of the limitations of this study is that urine S100 proteins were not compared with other emerging urinary biomarkers for LN activity; however, multiple S100 proteins were analyzed in both serum and urine to provide a more comprehensive review of this protein family in relation to cSLE. Despite the limitation to two time points per patient in our longitudinal cohorts, we were able to establish that improvement in LN activity corresponds to decreasing urine S100 levels. A validation cohort is needed to establish that these results can be replicated, because this was a pilot study with a limited sample size. However, given that this was a multicenter study with a diverse patient population, these results are more widely applicable than if the study had been done in a single center. Additionally, our control biopsy specimens for IHC were not ideal, given the large age difference from our patients with LN; however, most healthy children are not biopsied, making an age-matched and truly healthy control sample a rarity in our pathology sample bank.

## Conclusions

The results of this study demonstrate that urine S100A4 levels are a promising noninvasive biomarker for LN activity. The longitudinal cohort data showed that urine S100A4 levels are associated with changes in LN activity, with significantly decreased levels upon disease activity improvement in cSLE. Further studies are necessary both to better understand the origin of elevated S100A4 protein in LN and also to confirm these findings.

## Additional files

Additional file 1: Table S1. Demographic and clinical data on patients with cSLE with unpaired serum and urine samples from Cohorts X_s_ and X_u_. (DOCX 17 kb)

Additional file 2: Table S2. Median S100 and laboratory values in patients with cSLE from Cohorts L_s_ and L_u_. (DOCX 14 kb)

## Abbreviations

Cohort L_s_: Longitudinal serum cohort
Cohort L_u_: Longitudinal urine cohort
Cohort X_s_: Cross-sectional serum cohort
Cohort X_u_: Cross-sectional urine cohort
cSLE: Childhood-onset systemic lupus erythematosus
dsDNA: Double-stranded DNA
eGFR: Estimated glomerular filtration rate
EMT: Epithelial-mesenchymal transition
IHC: Immunohistochemistry
IRB: Institutional review board
ISN/RPS: International Society of Nephrology/Renal Pathology Society
LN: Lupus nephritis
mRNA: Messenger RNA
NET: Neutrophil extracellular trap
NGAL: Neutrophil gelatinase-associated lipocalin
NIH-AI: National Institutes of Health activity index
SLEDAI-2K: Systemic Lupus Erythematosus Disease Activity Index 2000
SLEDAI-R: Systemic Lupus Erythematosus Disease Activity Index 2000 renal domain
