# Supplementary material for: Urine S100 proteins as potential biomarkers of lupus nephritis activity
Source: Arthritis Res Ther. 2017 Oct 24;19:242. doi: 10.1186/s13075-017-1444-4 (PMC5655804; doi:10.1186/s13075-017-1444-4)
Supplement: Supplementary file 1 — Demographic and clinical data on patients with cSLE with unpaired serum and urine samples from Cohorts Xs and Xu. (DOCX 17 kb) [file 13075_2017_1444_MOESM1_ESM.docx]

| **Additional file 1: Table S1.** Demographic and clinical data on cSLE patients with unpaired serum and urine samples from Cohorts X_s_ and X_u_ | | | | |
| --- | --- | --- | --- | --- |
|  |  |  |  |  |
|  | **Cohort X_s_ (n = 100)** | | **Cohort X_u_ (n = 96)** | |
| **Characteristic** | **Active serum (n = 75)** | **Low disease activity serum (n = 25)** | **Active urine (n = 70)** | **Low disease activity urine (n = 26)** |
| **Age at diagnosis (years)** | 15 (12-19) | 13 (9-15) | 14 (12-16) | 13 (12-14.8) |
| **Disease duration (years)** | 5 (2-10) | 8 (3-14) | 2 (0-5) | 2.5 (1-6.8) |
| **Gender (female); n (%)** | 68 (90.7) | 25 (100) | 58 (82.9) | 24 (92.3) |
| **Race (white); n (%)** | 41 (54.7) | 17 (68) | 33 (47.1) | 10 (38.5) |
| **Ethnicity (hispanic); n (%)** | 31 (41.3) | 9 (36) | 6 (8.6) | 1 (3.8) |
| **Total SLEDAI-2K score** | 12 (9-16) | 2 (0-2) | 13 (10-19) | 2 (0-2) |
| **Active renal involvement; n (%)** | 41 (54.7) | 0 (0) | 52 (74.3) | 0 (0) |
| **Biopsy proven; n (%)** | *23 (92) | N/A | 50 (96.2) | N/A |
| **SLEDAI-R score** | 8 (4-12) | N/A | 8 (7-12) | N/A |
| **ISN/RPS Class of LN (III/IV/V); n (%)** | *1/11/10 (4.3/47.8/43.4) | N/A | 8/21/17 (16/42/34) | N/A |
| **eGFR < 75 mL/min/1.73m^2^; n (%)** | 2 (8) | N/A | 4 (8) | N/A |
| **Active extrarenal involvement; n (%)** | 72 (96) | 14 (56) | 68 (97) | 17 (65.4) |
| **Medications; n (%)** |  |  |  |  |
| **Oral or IV steroids** | 64 (84.2) | 12 (48) | 59 (84.3) | 13 (50) |
| **Hydroxychloroquine** | 61 (81.3) | 20 (80) | 57 (81.4) | 25 (96.2) |
| **Other immunosuppressant** | 38 (50.7) | 6 (24) | 43 (61.4) | 12 (46.2) |
| **Laboratory tests** |  |  |  |  |
| **Positive anti-dsDNA; n (%)** | *26 (57.8) | *4 (8.9) | 44 (62.9) | 5 (19.2) |
| **C3 (mg/dL)** | *86.8 (62.2-110) | *112 (93-120) | 77 (51.1-104) | 112 (92-127.5) |
| **C4 (mg/dL)** | *10.5 (7.5-15) | *15 (12-18) | 9.2 (5.7-15.4) | 16.7 (12.2-22.8) |
| **Random urine protein/creatinine** | *0.37 (0.2-1.7) | *0.13 (0.08-0.2) | 1.53 (0.2-3.6) | 0.1 (0.07-0.15) |
| **Active urinary sediment; n (%)** | *19 (42.2) | *0 (0) | 43 (61.4) | 0 (0) |
| * Patients with active LN and active extrarenal SLE only are combined into the active categorization in this table | | | | |
| * All table values are expressed as median (interquartile range) for continuous variables and number (percent) for categorical variables | | | | |
| * (*) Note that lab data whether LN biopsy-proven, ISN/RPS class of LN and eGFR for SLE patients with serum only available for CCHMC patients (n = 45), not Brazilian patients (n = 30). Percent in table calculated from patients with available values for these characteristics. | | | | |
