# Supplementary material for: Urine S100 proteins as potential biomarkers of lupus nephritis activity
Source: Arthritis Res Ther. 2017 Oct 24;19:242. doi: 10.1186/s13075-017-1444-4 (PMC5655804; doi:10.1186/s13075-017-1444-4)
Supplement: Supplementary file 2 — Median S100 and laboratory values in patients with cSLE from Cohorts Ls and Lu. (DOCX 14 kb) [file 13075_2017_1444_MOESM2_ESM.docx]

| **Additional file 2: Table S2.** Median S100 and laboratory values in cSLE patients from Cohorts L_s_ and L_u_ | | | | | |  |
| --- | --- | --- | --- | --- | --- | --- |
| **Median S100 or lab value** | **Cohort L_s_ (n = 47)** | | | **Cohort L_u_ (n = 39)** | | |
|  | **Active** | **Improved** | **p-value** | **Active LN** | **Improved LN** | **p-value** |
| **S100A4 (ng/mL)** | ND | ND | N/A | 7.4 (1.7-22.7) | 0.97 (0.5-2) | <0.0001 |
| **S100A6 (ng/mL)** | 126 (91-270) | 179 (90-301) | 0.4051 | 104 (39-193) | 50.7 (33-79) | 0.0075 |
| **S100A8/9 (ng/mL)** | 3991 (1147-7857) | 2712 (983-5216) | 0.1824 | 2620 (286-6365) | 865 (113-3857) | 0.019 |
| **S100A12 (ng/mL)** | 169 (80-383) | 161 (56-309) | 0.3746 | 12.2 (1.5-60) | 1.9 (0.3-9.7) | 0.0143 |
| **C3 (mg/dL)** | 83.6 (59.5-114.8) | 92.7 (72.1-117) | 0.1048 | 65 (45.4-93.3) | 95.1 (80.9-120.8) | 0.0003 |
| **C4 (mg/dL)** | 10.6 (7-16.2) | 15.3 (9.3-23.8) | 0.0167 | 8.6 (4.5-11.1) | 17.1 (12.5-24.5) | 0.0002 |
| **Random urine protein/creatinine** | 0.26 (0.1-1.79) | 0.12 (0.1-0.2) | 0.0011 | 2.1 (1.1-5.3) | 0.16 (0.1-0.3) | <0.0001 |

- All table values are expressed as median (interquartile range)
- The Wilcoxon signed-rank test was applied to analyze the change in S100 or lab value between paired samples
